# Medical Statistics, Their Force and Fallacy

**Published:** 1847-07-01

**Authors:** 


### MEDICAL STATISTICS, THEIR FORCE AND FALLACY. By *James Duncan*, A.M.,M.D. 8vo. p. 42. Dublin, 1847.

INTRODUCTORY lectures are proverbial for their dullness, their repetitions, and the inordinate quantity of inanities they are the privileged medium for the communication of. Dr. Duncan has thought proper to break through the ordinary routine, and the consequence is the production of a very interesting exposition of some of the deductions derivable from the examinatian of the Registrar-General's and the British Army Reports, and of some of the errors to which an undue reliance upon figures may give rise to.

Dr. Duncan first adverts to *the different degrees in which the two sexes are affected by disease.* It is well known that a larger portion of males *are born.* The English (1838-41) Registrar and Irish Census (10 years) Returns, give 2,240,938 male for 2,142,272 female births, and yet at any period the actual number of the sexes living is reversed. The general population of Great Britain and Ireland in 1841 was 13,097,012 males and 13,742,873 females. This result can only occur from the the excess of mortality in males, and the Reports above referred to register 1,318,853 male to 1,241,207 female deaths. Moreover, the difference does not occur at the advanced periods of life when the different habits of life might to some extent explain it, but in infancy, when every influencing condition of the sexes seems similar; so that for 406,684 male deaths under two years, there were but 344,935 females. After this period the sexes remain very similarly circumstanced until the period of puberty, when the difference is found to be in favour of the males until the 40th year. To about the 45th the deaths are nearly equal, after which until about 60, the males again form the excess—females constituting the majority of those who die in advanced years.

Dr. Duncan draws attention also to the *relative frequency of consumption in men and women.* Authors have been divided upon this point: but the Registrar's Report for 1838—40 gives us 90,611 deaths in a female population of 7,885,615, and 80,550 deaths in a male population of 7,668,245; but it is a curious fact, that while consumption is more frequent among *females* in the *general* and *rural* population, it is more frequent among *males* in *cities* and *large towns;* for while in 100,000 of the general population there would be but 378 male and 408 female deaths, in that of London there would be 451 males to 377 females, and in Birmingham 526 males to 410 females.

We cannot follow the author in his examination of the proportion of deaths caused by consumption in our military service in different parts of the world, important as information derived from such considerations is in confirming or correcting the prevalent ideas upon the effects of climate in this dreadful disease; and we must content ourselves by very briefly adverting to some of his observations upon the abuse of figures. For example, the superiority of any given institution in the number of cures it produces should never be affirmed until the rules governing the admission, diet, &c., of patients are understood. The ratio of mortality without this may be apparently, but not really, much greater in a given disease in a workhouse than in a hospital, or in two institutions ostensibly similar. The author agrees with those who regard the numerical method as incompetent to aid in the treatment of disease.

"A number of cases of a disease, say delirium tremens, are treated in a particular way, and the result is compared with an equal number of cases treated differently, and a conclusion is drawn in favour of the plan which appears to secure the largest number of recoveries out of the entire. To this theory as thus explained there are many objections. Let us suppose, however, that the trial has been made: to what practical result, I ask, can it possibly lead? Are we to understand that, having found out a certain plan to be successful in a large majority of cases of any disease, that it is to be applied, with unbending exactness, to every other example of it we may meet with? Is there to be no regard to the stage of the disease, the constitution of the patient, the character of the prevailing epidemic? If there is, of what value is the supposed statistical announcement, to guide us in the varying circumstances in which we may be placed? If such distinctions are disregarded, in what is our conduct less absurd than that of the mere pretender to physic, who, with a boldness only unequalled by the credulity of the public, invents his specific, and declares that it is a panacea for all diseases—that it is suited to all constitutions, and that, while it will infallibly cure, it will do no harm."

